# Analysis-ready optical underwater images of Manganese-nodule covered seafloor of the Clarion-Clipperton Zone

**DOI:** 10.1038/s41597-023-02245-5

**Published:** 2023-05-25

**Authors:** Benson Mbani, Jens Greinert

**Affiliations:** 1 DeepSea Monitoring Group, GEOMAR Helmholtz Center for Ocean Research Kiel, Wischhofstraße 1-3, 24148 Kiel, Germany; 2 Institute of Geosciences, Kiel University, Ludewig-Meyn-Str. 10-12, 24118 Kiel, Germany

**Keywords:** Ocean sciences, Computer science

## Abstract

We provide a sequence of analysis-ready optical underwater images from the Clarion-Clipperton Zone (CCZ) of the Pacific Ocean. The images were originally recorded using a towed camera sledge that photographed a seabed covered with polymetallic manganese-nodules, at an average water depth of 4,250 meters. The original degradation in visual quality and inconsistent scale among individual raw images due to different altitude implies that they are not scientifically comparable in their original form. Here, we present analysis-ready images that have already been pre-processed to account for this degradation. We also provide accompanying metadata for each image, which includes their geographic coordinates, depth of the seafloor, absolute scale (cm/pixel), and seafloor habitat class obtained from a previous study. The provided images are thus directly usable by the marine scientific community e.g., to train machine learning models for seafloor substrate classification and megafauna detection.

## Background & Summary

The recent advances in underwater optical imaging technologies have allowed for rapid acquisition of high-resolution images of the seabed across both temporal and spatial scales^1^. These images are valuable to marine scientists, as they provide for non-invasive monitoring and characterization of seafloor habitats, as well as quantification of the abundance and diversity of megafauna^2^. Images can be used for these purposes alone, or as a complementary dataset to verify and ground-truth acoustics-based marine habitat mapping^3^. Despite their usefulness, underwater optical images usually suffer from degraded visual quality due to the effects of light scattering, absorption and attenuation of (artificial) light as it propagates through the water column^1^. Collectively, these effects degrade the overall visual appearance of the images e.g., through poor contrast, greenish or blueish haze, and also gradual reduction in image brightness towards the edges of the image^4^. In addition to these degradations, the inability of in particular towed camera platform to maintain a consistent altitude above the seafloor further results in images that suffer from uneven scene brightness. This variation in altitude also causes the scale of each image (in pixels/centimeter) to vary, which implies that individual images do not represent the same spatial footprint on the seafloor^5^, and therefore cannot be semantically compared. Raw images thus need to be transformed prior to being used for scientific analysis. Performing these transformations can be both time-consuming and compute-intensive because of the huge volumes of high-resolution images that are nowadays acquired during scientific expeditions^6^.

In contrast to that, the analysis-ready images that we provide have undergone the necessary transformations as well as technical validations. The applied transformations include: correction for illumination drop-off from the center of the image towards the edges; local contrast enhancement that is necessary to equalize the distribution of pixel values of the image, so as to occupy the entire range of possible intensity levels; color normalization that corrects for uneven scene brightness among individual images by matching their intensity histograms; and finally, standardization of both the scale and visual footprints. Therefore, the images can be directly used in scientific research workflows e.g. to monitor seafloor geology, marine ecosystems, and megafaunal communities^2^.

**Fig. 1:**
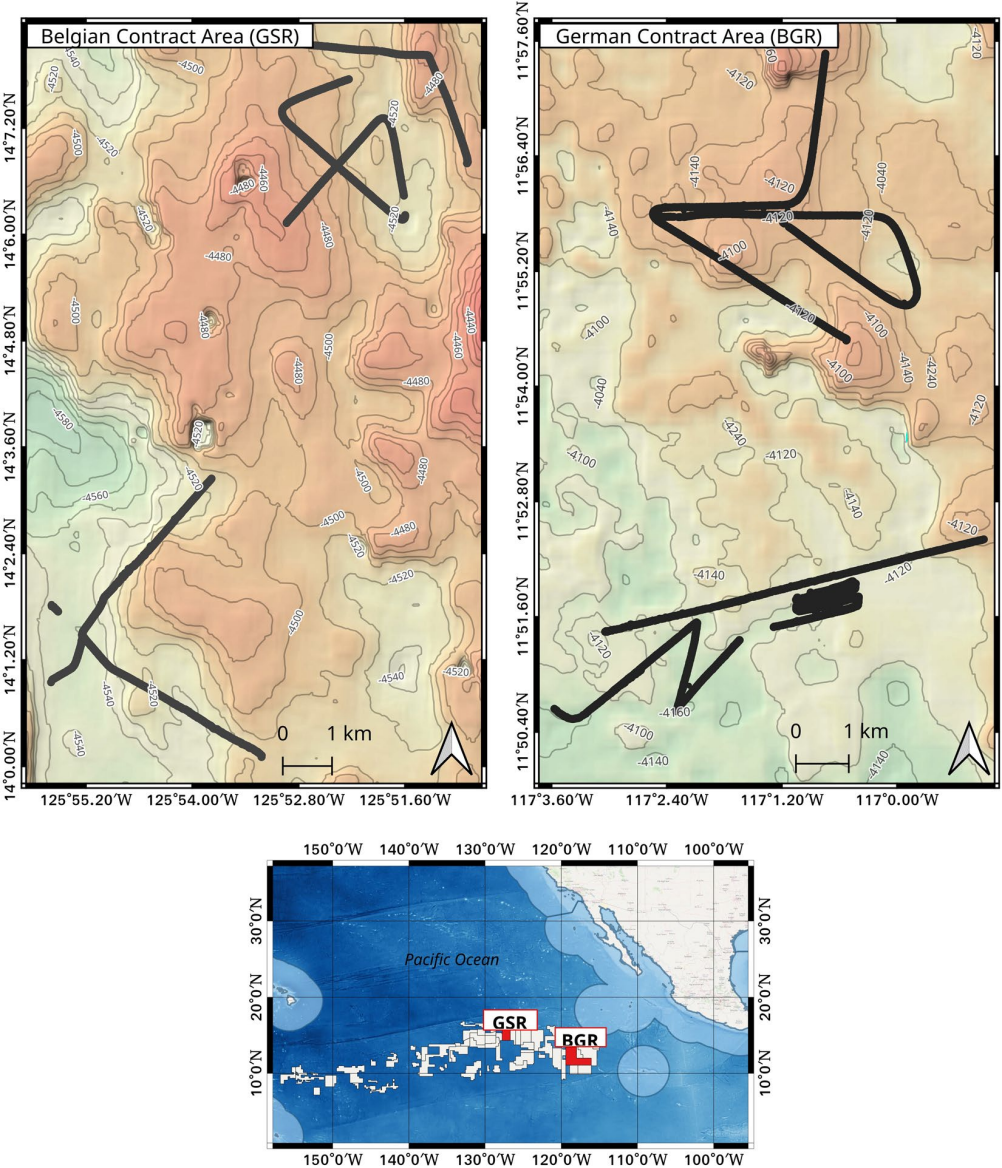
Map showing the camera deployment tracks during SONNE cruise SO268 to both the German and Belgian contract areas within the Clarion-Clipperton Zone of the Pacific Ocean.

The provided images were acquired during an expedition to the German and Belgian contract areas for Manganese-nodule exploration in the Clarion-Clipperton Zone (CCZ) of the Pacific Ocean, in the year 2019. The expedition was executed on board the German research vessel SONNE during cruise SO268, which was part of the MiningImpact project whose overarching scope was to quantify the impacts of potential polymetallic Manganese-nodule mining on the marine ecosystem^7^. Twelve video transects were undertaken within the two contract areas (Fig. 1), from which both video and still image frames were acquired. These raw images have been archived and published in PANGAEA^8^. We used these raw images in a previous study that aimed to develop an automated image-based workflow for semantic seafloor substrate classification; the findings of the study have been published in Mbani *et al*.^9^. As part of the data pre-processing workflow of the above-mentioned study, intermediate images were generated to be used in the main seafloor substrate classification task. These are the analysis-ready images (and associated metadata) that we contribute here to the scientific community.

## Methods

### Raw images

Below, we briefly present the methods that were applied during the acquisition, curation and archival of the raw image dataset^8^. These methods have been adopted from the SONNE SO268 cruise report^7^, as well as from the GEOMAR data management guidelines, whose technical details are documented by Schoening *et al*.^10^.

#### Acquisition

The raw images^8^ were acquired using a towed Ocean Floor Observation System (OFOS), which is an imaging platform comprising a steel frame that houses the camera and other sensors. The OFOS was towed at a speed of approximately 0.5 knots, while maintaining a data link to the ship through a fibre optical cable. A Canon EOS 5D Mark IV camera equipped with a 24 mm lens was used to record still images with a resolution of 30 megapixels, at a frequency of 0.1 Hz. The camera field of view was artificially illuminated by a set of strobe and LED lights. The position of the OFOS was tracked using a USBL underwater navigation system. To determine the scale of each photo (in pixels/centimeter), three laser pointers positioned around the camera projected red laser beams vertically downwards towards the center of each photo. This allowed estimating the ratio between the photographed laser separation distance (in pixels) to the actual distance (in centimeters).

#### Curation

After acquisition, the images were downloaded from the hard disk that was located in the pressure housing of the OFOS, and copied onto local hard drives. The curation process then involved renaming the images in a way that basic metadata information about each image could be accessed from the assigned file name. The navigation information from the USBL was quality controlled by first removing outliers, and then imputing missing entries using spline interpolation. Thereafter, each image was georeferenced by first parsing its file name to retrieve the acquisition time, and then using this time to index into the navigation file so as to retrieve the position information. Finally, the curated images were organized into folders that grouped images by deployment station, and backed up into separate Network Access Storage (NAS) drives, ready to be delivered back to the office after the expedition.

#### Data management

The post-cruise data management involved copying the curated images from the NAS drives to the GEOMAR’s in-house repository called ProxSys, which is a centralized media server that facilitates controlled data access, versioning and overall management. In addition, the curated images were also copied to the BIIGLE portal, which is a web-based platform that allows for collaborative image annotation among domain scientists^11^, and which is open to the public upon registration. Finally, the curated images were pushed to PANGAEA^8^, which is a world data center that allows for long term data archiving, publishing and reuse^12^.

### Analysis-ready images

Below, we describe the series of transformations we applied to the raw images, before they were ready to be used for characterizing the seafloor habitat. The transformations described are part of the automated image-based seabed classification workflow that is presented comprehensively in Mbani *et al*.^9^.

#### Light cone correction

This transformation was applied to account for the reduction in image brightness from the center of the image radially towards the edges. This illumination drop-off is usually caused by the perspective geometry of the artificial light source, in which the circular plane of the camera’s conic view volume that intersects the seafloor is illuminated strongly, whereas the intensity of illumination reduces towards the edges. As a result, the image edges appear dark, which limits the quality and quantity of information that can be inferred from these regions. To address this, we used the z-score normalization transformation, where the images were first sorted sequentially based on their acquisition time, and split into batches containing 50 images each (due to memory constraints). Considering images within each batch, the transformation involved pixel wise subtraction of the mean, followed by pixel wise scaling to unit variance. This transformation reduced the effect of the light cone by ensuring that all the pixels had a common origin in feature space, and that the range over all the dimensions of this feature space had a standard deviation of one.

#### Contrast enhancement

We applied adaptive histogram equalization transformation to maximize the contrast of the light-cone corrected images. This improved the image contrast by ensuring that the distribution of pixel intensities within local image regions is as uniform as possible. This in turn improves the global image contrast, since the pixel values now occupy the entire range of available intensity values, instead of peaking over a narrow range.

#### Color normalization

The variation in the altitude of the OFOS above the seafloor caused uneven scene brightness and color among the acquired images. We addressed this problem by choosing a reference image with good overall scene brightness and color values, and then equalized the intensity distribution of all the other images (channel wise) relative to this reference image, resulting in color normalized images. We chose the reference image to be the one with the maximum resolution closest to the seafloor.

#### Standardization of spatial footprint size

The varying altitude of the camera platform also caused individual images to have inconsistent scale and spatial footprint. We addressed this problem by first calculating the scale of each image (in pixels/centimeter), and then rescaling all the images relative to the median scale. Finally, we center cropped the rescaled images to a standard footprint size of 1.6 square meters, which was the minimum over all the rescaled images. The resulting images are then ready for direct use e.g., for machine learning models aimed at seafloor habitat classification (see usage notes section of this paper).

We point out here that the center cropping reduced the size of the images by 50% in both height and width. Therefore, standardization of spatial footprint might not be necessary (so important) for images that were recorded from a camera platform at a constant altitude e.g., some AUVs.

**Fig. 2:**
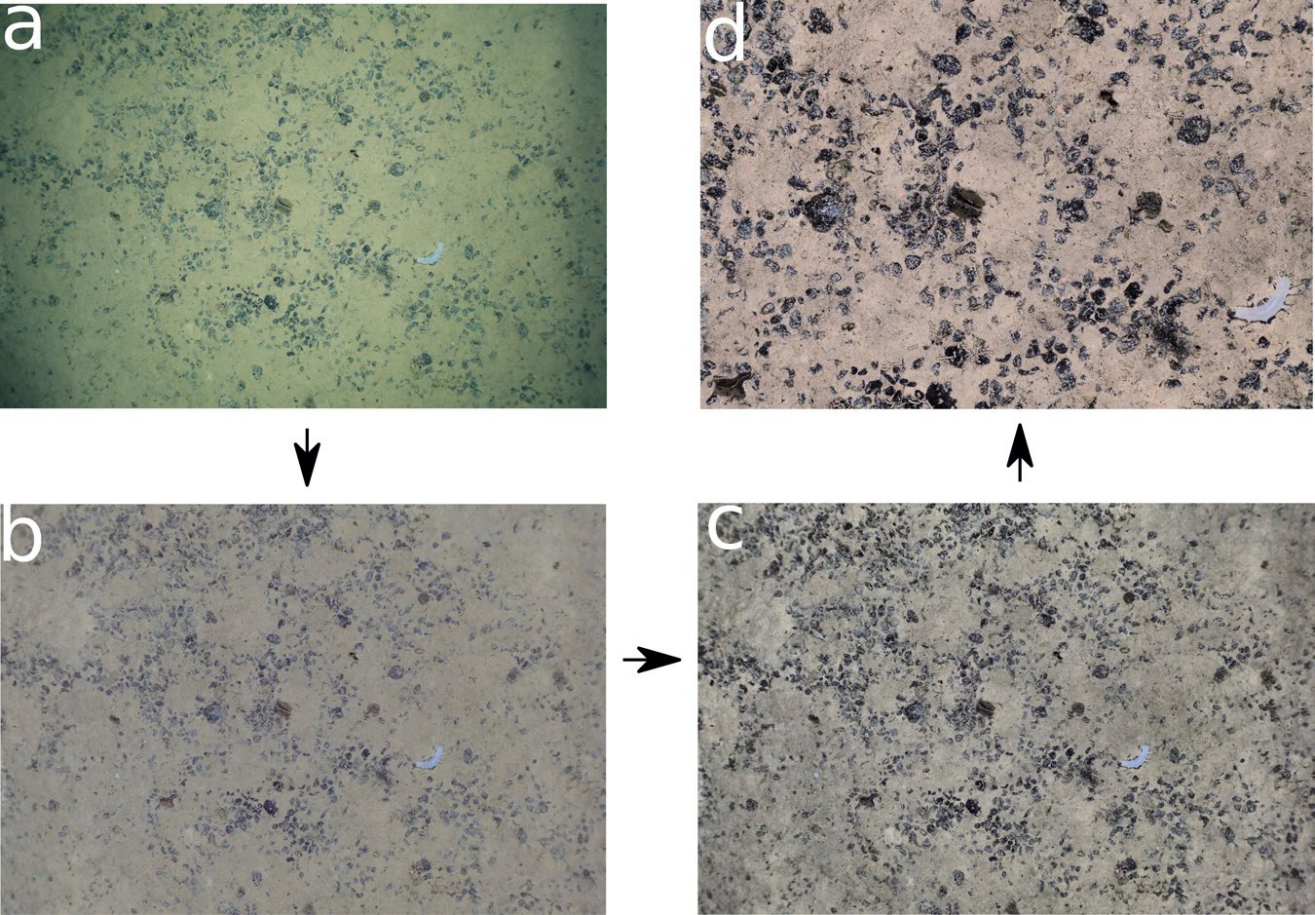
Visualization of the transformations applied to an example original image to obtain the final analysis-ready image. The transformed images are positioned counter-clockwise: (**a**) Original image (**b**) Light cone corrected image (**c**) Contrast enhanced image (**d**) Color normalized image with standardized footprint size of 1.6 square meters.

Figure 2 shows an example image that is being processed through each of the above-described transformations to generate the final analysis-ready image that we provide in this paper.

## Data Records

### Images

40,476 analysis-ready optical underwater images from the Clarion-Clipperton Zone have been archived and published in PANGAEA^13^. These images are available for immediate download as color normalized JPG files of size 1.5 MBytes. Each image has 2,240 rows and 3,360 columns of pixels, which corresponds to a standardized spatial footprint of approximately 1.6 square meters on the seabed. The scale of each image has also been standardized to 21.5 pixels/centimeter, which allows for a consistent conversion of measurements from pixels to real world units (e.g., meters). The file naming convention and other metadata allows users to select subset(s) of the images they need (see the adopted naming convention in the metadata section below); they could either download the entire dataset, specific dives, or images from a particular contract area.

### Metadata

To complement the images, we also provide their corresponding metadata as a separate csv file. These metadata include:

The image name which follows the file naming convention: <*cruise_station_platform_date_time.JPG*>.

The contract/license area from which the image was acquired, which could either be Belgian or German.

The water depth (meters) at which each image was acquired; images acquired during camera deployment at a specific station have the depth value of that station.

Geographic coordinates in latitude and longitude with coverage defined (in decimal degrees) as follows: median latitude 12.608845, median longitude −119.975256, south-bound latitude 11.842236, west-bound longitude −125.926561, north-bound latitude 14.136652, and east-bound longitude −116.984906.

Date and time of acquisition (up to seconds in resolution) with coverage starting from 2019-03-04T09:35:10 all the way until 2019-05-10T09:53:13. The acquisition time of the provided images is referenced to the Coordinated Universal Time (UTC), and exactly matches the acquisition time of the corresponding original images^8^. Therefore, users who would like to relate our analysis-ready images with the corresponding original versions should use the date/time attribute instead of the respective file names. This is because the file naming convention may vary depending on the user (or organization), but the acquisition time is a property of the image that does not change, and is therefore a persistent unique identifier.

Original scale of each image in units of centimeters/pixel as obtained from the automatic laser point detection workflow^9^. This workflow was applied to the original images in order to automatically detect red laser points that were projected vertically on the seafloor during image acquisition; these laser points were visible in both nodules and mud/sediment. The scale was then determined as the ratio between the distance separating the detected laser points (in pixel units) and their known calibrated distance (in centimeters). Whereas the scale of the original images varied depending on the altitude of the imaging platform, the analysis-ready images that we provide here have already been standardized to have a mean scale of 21.5 pixels/centimeter.

**Fig. 3:**
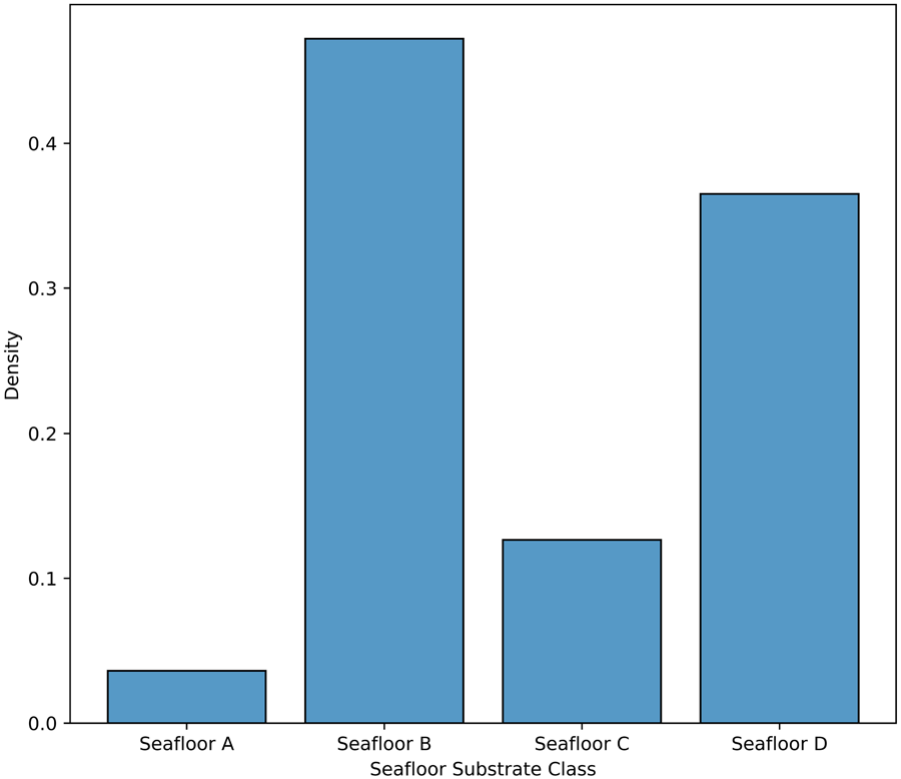
Histogram showing the distribution of seafloor classes. Seafloor B comprising patchy nodules had the highest frequency distribution, whereas few images were classified as Seafloor A because it was artificially created by plough marks and subsequent settling plume from the dredging experiment.

The assigned seafloor substrate class of each image. This is based on the previously published automated seafloor classification workflow^9^, which trained a convolution neural network to classify each image into one of the following seafloor classes: *Seafloor A* that represents a seabed that is predominantly covered with turned-over sediment blanket or plough marks, such that none or few Mn-nodules are visible in the image; *Seafloor B* comprises a seabed covered by patchy Mn-nodules that only partly cover the seabed; *Seafloor C* is characterized by Mn-nodules whose spatial distribution can be described as densely distributed per unit area; Finally, *Seafloor D* comprises a seabed covered with Mn-nodules that are qualitatively larger in size relative to those in all the other classes. In addition to these classes, the classification score for each image that represents the confidence of the classifier is also provided in the metadata file. The histogram showing the distribution of the seafloor substrate classes is shown in Fig. 3. We encourage interested readers to refer to the results section of Mbani *et al*.^9^ for qualitative examples of the above-described seafloor substrate classes, as well as the quantitative performance evaluation of the convolutional neural network classifier.

## Technical Validation

We performed technical validation on the provided analysis-ready images to quantify to which extent the applied transformations achieved the desired outcomes. Light cone correction transformation was only validated visually, since the analysis ready images were center cropped to standardize the spatial footprint size, and therefore their edges could not be quantitatively compared anymore with the raw images.

**Fig. 4:**
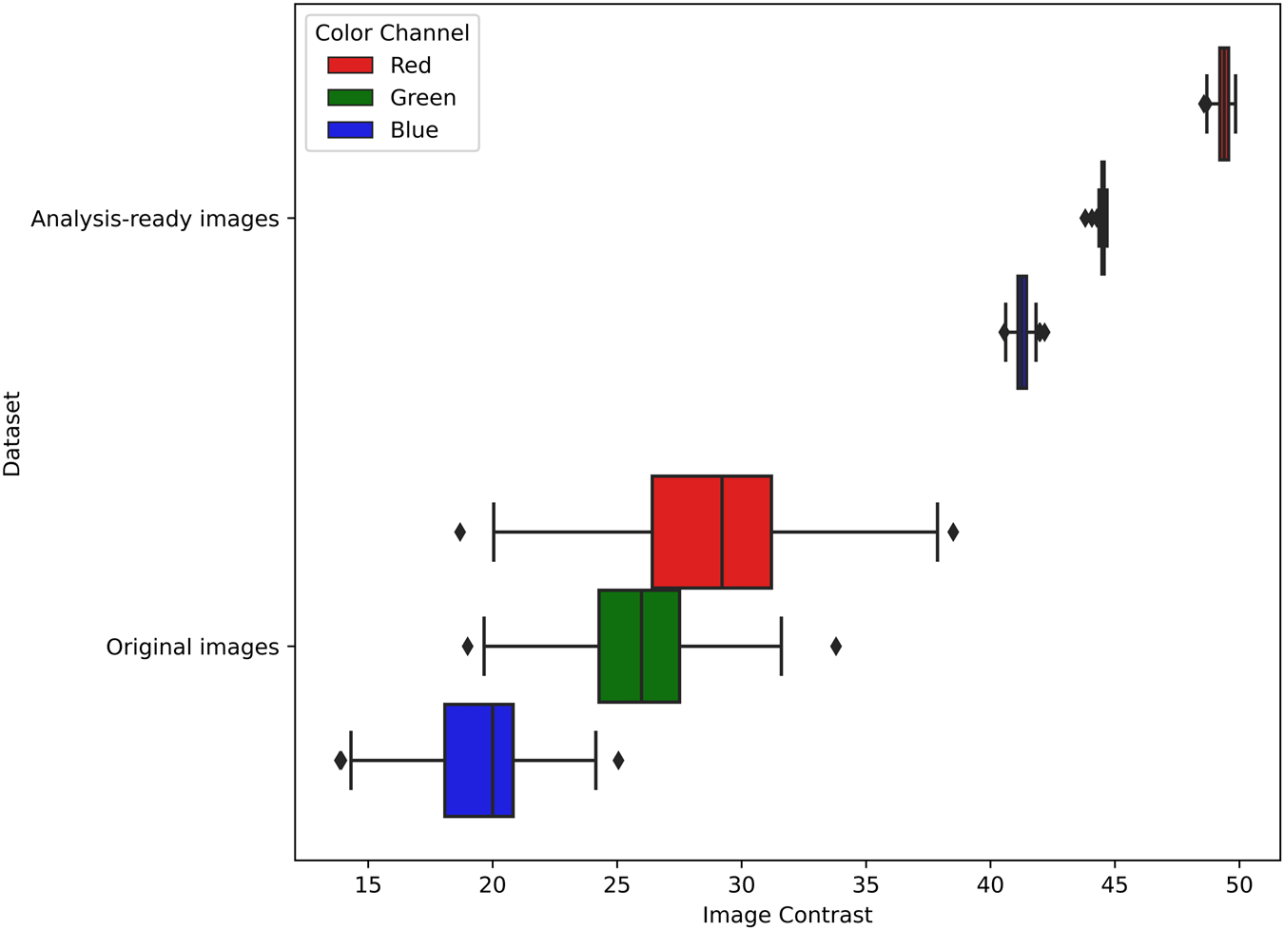
Evaluation of the improvement in image contrast among the analysis-ready images compared to the original images. Over each (R, G, B) color channel, original images had low contrast with a high variance, whereas analysis-ready images had consistently high contrast with low variance.

Contrast maximization was validated by comparing the contrast of the raw images against that of the analysis-ready images. The image contrast was quantified using the root mean square metric^14^, which is calculated as the standard deviation of the pixel intensities for each (*R, G, B*) channel; higher values of the metric indicate higher contrast. Our validation results in Fig. 4 show that compared to the raw images, the contrast of the analysis-ready images improved by a factor of 2 (averaged over all channels). This improved contrast was consistent among individual analysis-ready images, as indicated by the low variance of 0.1 in each color channel. Differently, the low contrast of the raw images still showed high variance among individual images (*R* = *14.7, G* = *7.1, B* = *5.0*), which implies that extracting visual features is more difficult.

**Fig. 5:**
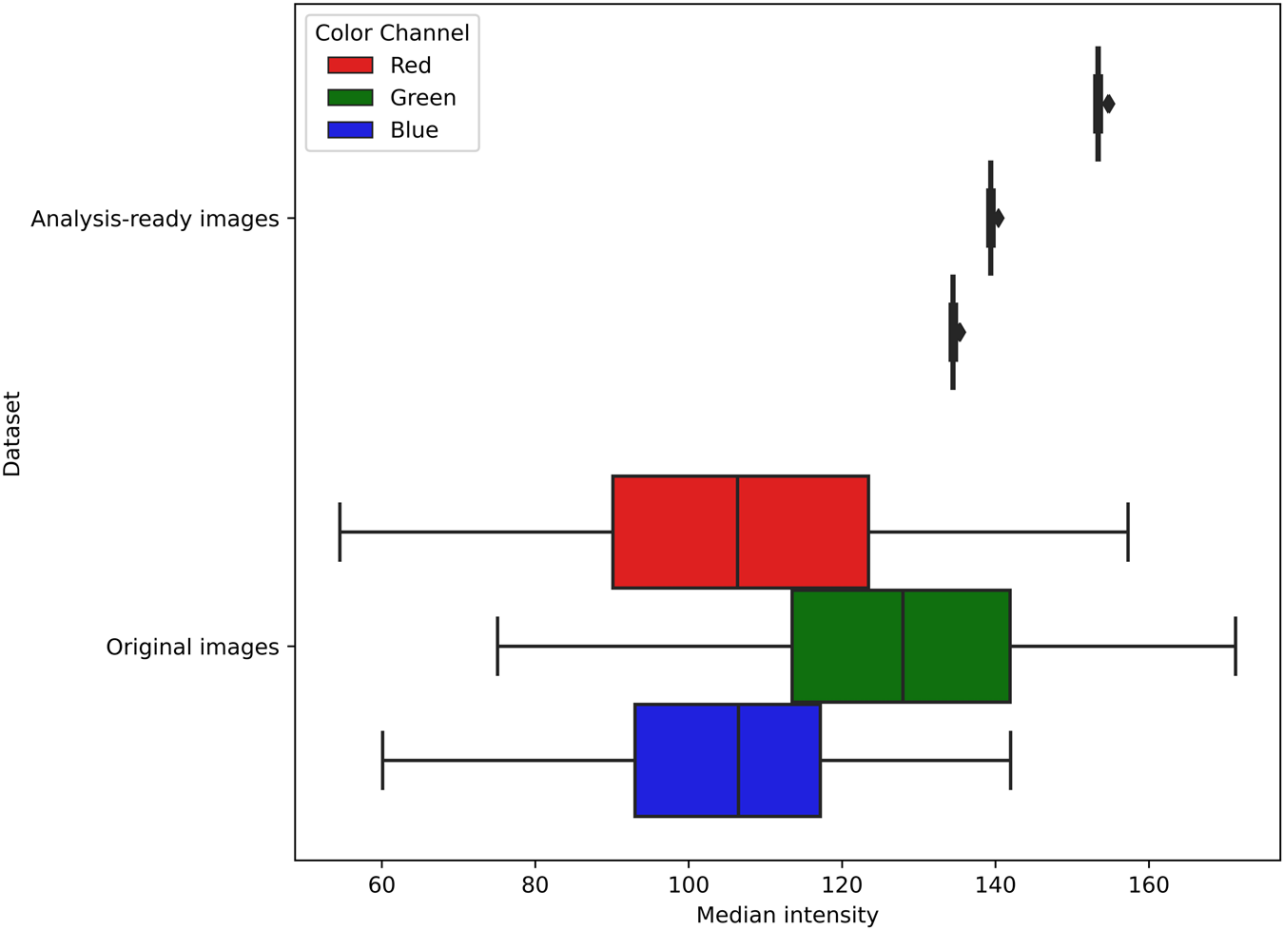
Evaluation of the normalization of image brightness among the analysis-ready images relative to the original images. The low variance in median intensity over each (R, G, B) color channel of the analysis-ready images indicates that their brightness is normalized. This is in comparison to the high variance in the intensity of the original images.

Color normalization was validated by comparing the brightness of the raw images against the analysis-ready images. The median intensity for each image was used as the metric for quantifying the scene brightness channel wise^15^. The validation results in Fig. 5 indicates that the median intensity of the raw images showed a high variance in each channel (*R* = *577.4, G* = *470.8, B* = *330.3*), which was the reason for the perceived uneven scene brightness. The analysis-ready images showed very low variance in median intensities across all channels (*R* = *0.07, G* = *0.04, B* = *0.04*), which implies that the overall scene brightness among the individual images is more consistent.

**Fig. 6:**
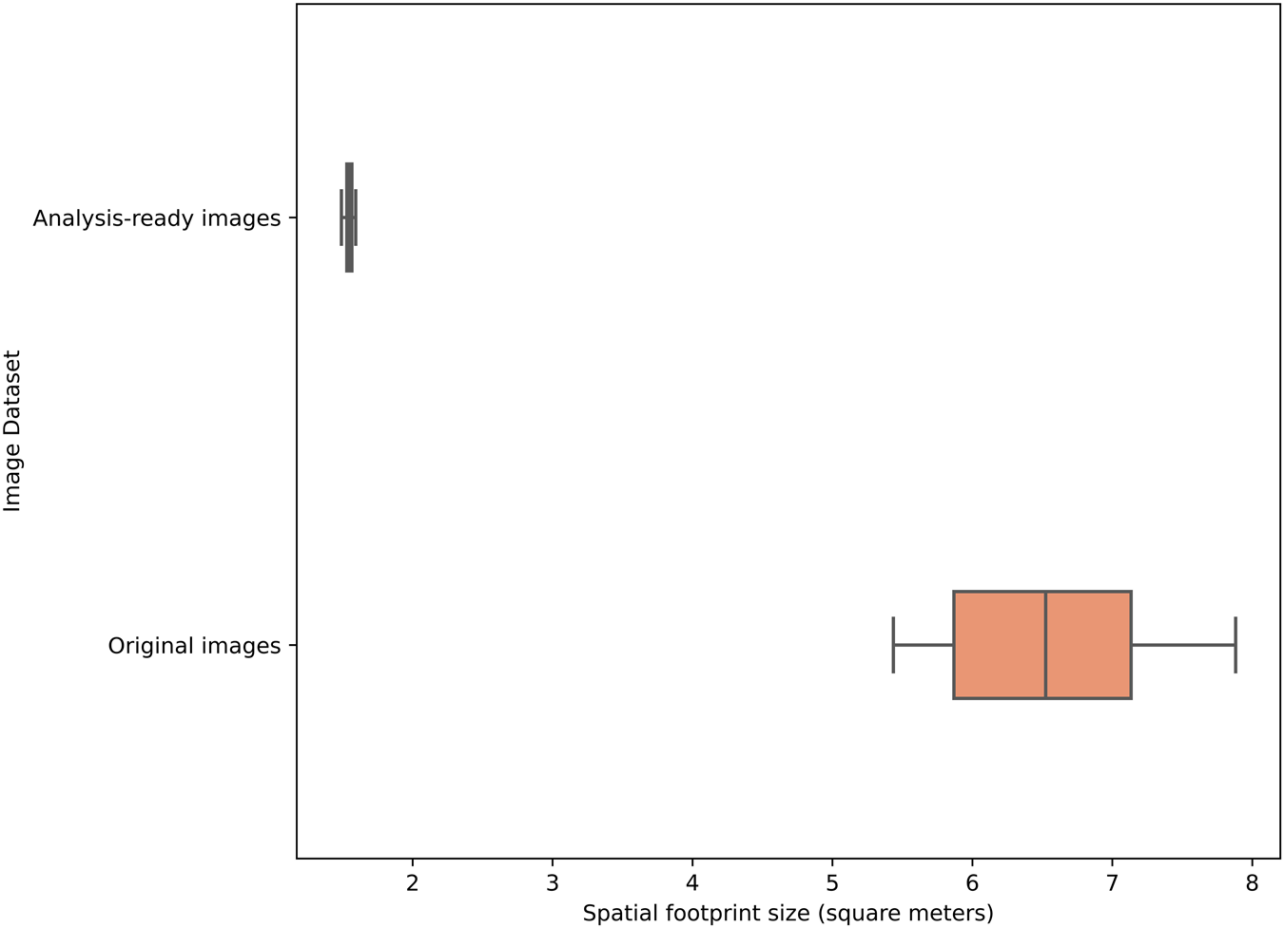
Evaluation of the standardization in spatial footprint among analysis-ready images relative to the original images. The high variance in the spatial footprints among original images implies that scientific information extracted from them cannot be semantically compared. On the other hand, the standardized spatial footprint among the analysis-ready images renders them useful e.g., as sampling units. Note the reduction in footprint size in the analysis-ready images as a result of center cropping.

Standardization of spatial footprint among analysis-ready images was validated by comparing their footprint size on the seafloor (in square meters) relative to the raw images. The footprints sizes were determined based on automatically detected laser points^9^, and further verified through manual inspection. Figure 6 shows that the variance in spatial footprint sizes among raw images was high (0.5) compared to the variance among the analysis-ready images (0.0005).

## Usage Notes

The results from the technical validation show that the analysis-ready images that we provide are directly usable for scientific analysis. Below, we describe a few examples of potential use cases.

### Seafloor substrate classification

This involves partitioning the seabed into a finite number of semantic seafloor habitat categories classes based on the interpretation of visual features extracted from the images. The classification can be achieved either by inviting domain experts to manually inspect and annotate each image with a habitat class, or by deploying a trained machine learning model.

The analysis-ready images are suitable for both of these approaches. On the one hand, the images can be uploaded to an annotation platform such as BIIGLE^11^, which has an intuitive web-based user interface that allows a team of annotators to seamlessly collaborate in assigning habitat labels to the images based on standardized annotation protocols^16^. Alternatively, a few example images can be annotated and used to train a machine learning model e.g., a random forest classifier. This trained classifier can then be used to automatically label the rest of the images, which is much more convenient and scalable compared to the purely manual approach. The results obtained from either of the approaches can then be used for domain-specific use cases e.g. to determine the type and density of Manganese-nodule coverage on the seafloor^9,17^, or validate acoustic-based seafloor substrate classification and mapping^18^.

### Megabenthic fauna detection

Understanding abundance and spatial distribution of megabenthic fauna is key towards conservation and management of marine ecosystems. Optical images allow for non-invasive monitoring of megabenthic fauna which can potentially span wide geographic extents. The provided analysis-ready images are directly usable as comparable sampling units for megafaunal community assessment studies. This assessment can be done either through the conventional manual identification and counting approaches, or through automation approaches that use state-of-the art object detection models^2,19^.

### Effect of sediment plume redeposition

Deep sea mining of marine resources e.g., polymetallic Manganese nodules has both economic and environmental implications. On the one hand, Mn-nodules contain significant concentrations of nickel, cobalt and copper, whose availability is needed for the energy transition from fossil fuels to low carbon emitting technologies^20^. On the other hand, the exploitation of these minerals involves large-scale dredging operations on the seabed, which re-suspends sediment into the near-bottom water. The subsequent redeposition of this sediment plume negatively affects the sensitive and slow growing fauna. The provided analysis-ready images contain survey tracks that were photographed before and after a sediment dredge experiment, and these could be used together with other sensor datasets to assess the spatial extent of this redeposition e.g. as was done by Peukert *et al*.^21^.

## Data Availability

The open-sourced code used for performing the light cone correction, contrast enhancement, color normalization transformations, as well technical validation can be accessed publicly through this online Gitlab repository^22^: (https://git.geomar.de/open-source/AI-SCW).
